# Regulation and functional role of the Runt-related transcription factor-2 in pancreatic cancer

**DOI:** 10.1038/sj.bjc.6603984

**Published:** 2007-09-18

**Authors:** H Kayed, X Jiang, S Keleg, R Jesnowski, T Giese, M R Berger, I Esposito, M Löhr, H Friess, J Kleeff

**Affiliations:** 1 Department of General Surgery, University of Heidelberg, Heidelberg, Germany; 2 Molecular Gastroenterology Unit, German Cancer Research Centre, Heidelberg, Germany; 3 Department of Medicine II, University of Heidelberg, Mannheim, Germany; 4 Institute of Immunology, University of Heidelberg, Heidelberg, Germany; 5 Unit of Toxicology and Chemotherapy, German Cancer Research Centre, Heidelberg, Germany; 6 Institute of Pathology, University of Heidelberg, Heidelberg, Germany

**Keywords:** pancreatic adenocarcinoma, stellate cells, osteonectin, matrix metalloprotease, transforming growth factor-*β*, tumour microenvironment

## Abstract

Recent evidence suggests that Runt-related transcription factors play a role in different human tumours. In the present study, the localisation of the Runt-related transcription factor-2 (Runx2), its transcriptional activity, as well as its regulation of expression was analysed in human pancreatic ductal adenocarcinoma (PDAC). Quantitative real-time PCR and immunohistochemistry were used for Runx2 expression and localisation analysis. Runt-related transcription factor-2 expression was silenced using specific siRNA oligonucleotides in pancreatic cancer cells (Panc-1) and immortalised pancreatic stellate cells (IPSCs). Overexpression of Runx2 was achieved using a full-length expression vector. TGF-*β*1, BMP2, and other cytokines were assessed for their potential to regulate Runx2 expression. There was a 6.1-fold increase in median Runx2 mRNA levels in PDAC tissues compared to normal pancreatic tissues (*P*<0.0001). Runt-related transcription factor-2 was localised in pancreatic cancer cells, tubular complexes, and PanIN lesions of PDAC tissues as well as in tumour-associated fibroblasts/stellate cells. Coculture of IPSCs and Panc-1 cells, as well as treatment with TGF-*β*1 and BMP2, led to increased Runx2 expression in Panc-1 cells. Runt-related transcription factor-2 overexpression was associated with decreased MMP1 release as well as decreased growth and invasion of Panc-1 cells. These effects were reversed by Runx2 silencing. In conclusion, Runx2 is overexpressed in PDAC, where it is regulated by certain cytokines such as TGF-*β*1 and BMP2 in an auto- and paracrine manner. In addition, Runx2 has the potential to regulate the transcription of extracellular matrix modulators such as SPARC and MMP1, thereby influencing the tumour microenvironment.

Runt-related transcription factor-2 (Runx2, also known as core-binding factor, runt domain, *α*-subunit 1, CBFA1, AML3, or OSF2) is the human homologue of mouse PEBP2A and acts as an osteoblast-specific transcription factor (Ogawa *et al*, 1993; Levanon *et al*, 1994). Runt-related transcription factor-2 has been isolated from haematopoietic cells and has a close homology to Runx1 and Runx3 (Ogawa *et al*, 1993). During embryonic development, Runx2 controls bone cell differentiation, and after birth, Runx2 controls bone matrix deposition, especially collagen I, by differentiated osteoblasts (Ducy *et al*, 1999). Homozygous Runx2 mutations are lethal perinatal (Komori *et al*, 1997), and altered chondrocyte morphology is observed in Runx2 heterozygous mice (Zheng *et al*, 2003). Runt-related transcription factor-2 acts as a scaffold that controls the integration, organisation, and assembly of regulatory factors for skeletal gene expression (Stein *et al*, 2004). Thus, Runx2 mutations cause cleidocranial and teeth dysplasia and altered endochondral ossification (Mundlos *et al*, 1997; D'Souza *et al*, 1999). This occurs via altered Runx2 regulation of hypertrophic chondrocyte-specific genes (Yoshida *et al*, 2004). In contrast, overexpression of Runx2 enhances osteoclast differentiation *in vitro* and bone resorption *in vivo*, and leads to overexpression of Rankl and collagenase-3 (MMP13) (Geoffroy *et al*, 2002).

Runt-related transcription factor-2 transcriptional activity is regulated by multiple pathways, such as the MAPK, PI3K, and STAT pathways (Lee *et al*, 2002; Xiao *et al*, 2002; Fujita *et al*, 2004). Runt-related transcription factor-2 also regulates several downstream target genes, such as extracellular matrix proteins, growth factors and receptors, mitochondrial proteins, and transcription factors (Gaikwad *et al*, 2001).

Runt-related transcription factor-2 regulation and transcriptional activity are linked with increased growth, invasion, and metastasis in breast cancer (Barnes *et al*, 2003), prostate cancer (Brubaker *et al*, 2003; Pratap *et al*, 2005), colorectal cancer (Wai *et al*, 2006), lymphoma, leukaemia and myeloma (Wotton *et al*, 2002; Castilla *et al*, 2004; Colla *et al*, 2005; Blyth *et al*, 2006).

Previously, a number of target genes for Runx2, such as SPARC (Guweidhi *et al*, 2005), MMP1 (Gress *et al*, 1995), IBSP (Kayed *et al*, 2006), and SPP1 (Kolb *et al*, 2005), have been shown to play a role in pancreatic carcinogenesis. In addition, a number of growth factors that have the potential to regulate Runx2 expression also play a role in pancreatic carcinogenesis, such as TGF-*β*1 (Friess *et al*, 1993a, 1993b; Yamanaka *et al*, 1993), BMP2 (Kleeff *et al*, 1999), and IHH (Kayed *et al*, 2004). Therefore, in the present study the localisation, transcriptional activity, and regulation of Runx2 expression in human pancreatic ductal adenocarcinoma (PDAC) was analysed.

## PATIENTS AND METHODS

### Tissue sampling

Pancreatic ductal adenocarcinoma (*n*=17) and chronic pancreatitis (CP; *n*=13) tissue specimens were obtained from patients in whom pancreatic resections were performed. Normal human pancreatic tissue samples (*n*=16) were obtained through an organ donor programme from previously healthy individuals. All samples were confirmed histologically. Freshly removed tissues were fixed in paraformaldehyde solution for 12–24 h and then paraffin embedded for histological analysis. In addition, a portion of human pancreatic tissue samples was preserved in RNAlater (Ambion Europe Ltd, Huntingdon, Cambridgeshire, UK), or snap-frozen in liquid nitrogen immediately upon surgical removal and maintained at −80°C until use. The Human Subjects Committee of the University of Heidelberg, Germany, approved all studies. Written informed consent was obtained from all patients.

### Quantitative real-time polymerase chain reaction

All reagents and equipment for mRNA/cDNA preparation were supplied by Roche Applied Science (Mannheim, Germany). mRNA of human pancreatic tissues was prepared by automated isolation using the MagNA Pure LC Instrument and Isolation kit I (for cells) and kit II (for tissues). cDNA was prepared using the First Strand cDNA Synthesis kit for RT–PCR according to the manufacturer's instructions. The primer sequences for all genes were obtained from Search-LC (Heidelberg, Germany). Real-time PCR was performed using the LightCycler FastStart DNA SYBR Green kit. The number of specific transcripts was normalised to housekeeping genes (cyclophilin B and HPRT) and presented as adjusted transcripts/*μ*l cDNA, as described previously (Kayed *et al*, 2004).

### Immunohistochemistry and immunocytochemistry

Paraffin-embedded tissue sections 2–3 *μ*m thick were deparaffinised in xylene and rehydrated in progressively decreasing concentrations of ethanol. Thereafter, the slides were placed in washing buffer (10 mM Tris-HCl, 0.85% NaCl, 0.1% bovine serum albumin, pH 7.4) and subjected to immunostaining. Antigen retrieval was performed by boiling tissue sections in 10 mM citrate buffer for 10 min in a microwave oven. To confirm the specificity of Runx2 staining, the sections were incubated with a rabbit polyclonal (Sigma-Aldrich, St Louis, MO, USA), a goat polyclonal (R&D Systems GmbH, Wiesbaden, Germany), or a rat monoclonal Runx2 antibody (R&D Systems GmbH). The corresponding normal IgGs were used as negative controls. The slides were then rinsed with washing buffer and incubated with HRPO-conjugated anti-rabbit (Amersham International, Buckinghamshire, UK), anti-goat (Santa Cruz Biotechnology Inc., Santa Cruz, CA, USA), or anti-rat IgG (Amersham International), respectively, for 1 h at room temperature. Tissue sections were then washed in washing buffer and DAB-chromogen/substrate mixture (DAKO, Hamburg, Germany) was applied to each section. Slides were analysed using the Axioplan 2 imaging microscope (Carl Zeiss Light Microscope, Göttingen, Germany). For immunocytochemistry, immortalised primary human pancreatic stellate cells (IPSCs) (Jesnowski *et al*, 2005) and Panc-1 cells were seeded on SuperFrost microscope slides (Menzel GmbH & Co KG, Braunschweig, Germany) overnight till adherent, and fixed with 3.5% paraformaldehyde for 25 min, and quenched with 30 mM glycine/PBS for 5 min. Permeabilisation of the cell membrane was carried out with 0.1% Triton X-100 for 5 min at room temperature. Immunostaining was then performed as described above using the goat polyclonal Runx2 antibody (R&D Systems GmbH). Slides were analysed using the Axioplan 2 imaging microscope (Carl Zeiss Light Microscope).

### Cell culture

Panc-1 pancreatic cancer cells and IPSCs were routinely grown in DMEM medium supplemented with 10% fetal calf serum (FCS) and 100 U ml^−1^ penicillin (complete medium), and incubated in a 5% CO_2_ humid atmosphere. For induction experiments, cells were seeded in 10 cm cell culture plates in 10% FCS growth medium and allowed to attach for 12 h. Growth medium was replaced by serum-reduced medium (1% FCS), and supplemented with recombinant TGF-*β*1 (500 pM), BMP2 (100 ng ml^−1^), FGF2 (10 ng ml^−1^), Shh (500 ng ml^−1^), Ihh (500 ng ml^−1^) (R&D Systems GmbH) and TNF-*α* (100 ng ml^−1^) (Promega Biosciences Inc., Mannheim, Germany) for 48 h. The doses were determined to ensure the efficacy and absent toxicity of each factor (Nakamura *et al*, 1997; Kleeff *et al*, 2000; Li *et al*, 2003; Kayed *et al*, 2004; Guo *et al*, 2006). Afterwards, cell culture supernatants, cell lysates, and mRNA were isolated as described. For coculture experiments without cell-to-cell contact, Panc-1 cells as well as IPSCs were seeded in both the wells and inserts of 12-well plates supplemented with permeable 0.4 *μ*m polyester membranes (Sigma-Aldrich) for 48 h.

### siRNA transfection

Panc-1 cells and IPSCs were grown in complete DMEM medium in 10 ml cell culture plates until 50% confluence. Runt-related transcription factor-2 siRNA transfection was performed using RNAiFect transfection reagent (Qiagen, Hilden, Germany) according to the manufacturer's instructions. Cells were transfected with 5 *μ*g siRNA (Runx2 target sequence AATGGCAGCACGCTATTAAAT) and control siRNA (target sequence AATTCTCCGAACGTGTCACGT) (Qiagen) for 48 h.

### Transient transfection of pancreatic cancer and stellate cells

The full-length cDNA encoding human Runx2 (pcDNA3.1/Runx2) was kindly provided by LD Quarles (Department of Medicine, Duke University Medical Center, Durham, NC, USA). Transient transfection of Panc-1 cells and IPSCs with the pcDNA3.1/Runx2 vector was carried out using the Lipofectamine method (Life Technologies, Karlsruhe, Germany) according to the manufacturer's instructions. Briefly, cells were seeded in 10 cm dishes in complete cell culture medium until 50% confluence. The medium was replaced by serum-free medium containing 5 *μ*g of pcDNA3.1/Runx2 or empty vector in transfection mixture for 3 h. An equal volume of medium containing 20% FCS was added for 24 h, and then replaced by complete medium.

### Enzyme-linked immunosorbent assay

Enzyme-linked immunosorbent assay (ELISA) kits were used for determining MMP1 protein levels in cell culture supernatants according to the manufacturer's instructions (RayBiotech Inc., Köln, Germany).

### Immunoblotting

Cells were washed twice with ice-cold PBS and lysed with lysis buffer (50 mM Tris-HCl, 100 mM NaCl, 2 mM EDTA, 1% SDS) containing one tablet of complete mini-EDTA-free protease inhibitor cocktail (Roche Applied Science). Protein concentration for both cell lysates and supernatant was determined by the BCA protein assay (Pierce Chemical Co, Rockford, IL, USA). Protein/lane (20 *μ*g) were separated on SDS-polyacrylamide gels and electroblotted onto nitrocellulose membranes. Membranes were then incubated in blocking solution (5% non-fat milk in 20 mM Tris-HCl, 150 mM NaCl, 0.1% Tween-20), followed by incubation with a rabbit polyclonal Runx2 antibody or a mouse monoclonal SPARC antibody (R&D Systems GmbH) at 4°C overnight. The membranes were washed and incubated with donkey anti-rabbit (Santa Cruz Biotechnology Inc.) or anti-mouse HRPO-conjugated IgG (Amersham International) for 1 h at room temperature. Equal loading and transfer was confirmed using a goat polyclonal *γ*-tubulin, or a rabbit polyclonal anti-ERK2 antibody (Santa Cruz Biotechnology Inc.), and Ponceau S staining (Sigma-Aldrich). Antibody detection was performed by an enhanced chemiluminescence reaction (Amersham International).

### Cell growth assays

Cells were seeded at a density of 5000 cells well^−1^ in 96-well plates for 48 h. Then, 10 ml MTT (5 mg ml^−1^) dissolved in PBS pH 7.4 were added to each well and incubated for 4 h at 37°C. Subsequently, cellular formazan was solubilised with 0.04 mM HCl/isopropanol. Optical density was measured at 570 nm with an ELISA plate reader (Opsys MR, ThermoLabsystems, Frankfurt, Germany). All assays were performed in triplicate.

### *In vitro* invasion assays

The Matrigel invasion assay (BD Biosciences, Heidelberg, Germany) was used to assess the invasive potential. Briefly, BioCoat Matrigel invasion chambers were rehydrated according to the manufacturer's instructions. Five hundred microlitres of DMEM cell culture medium supplemented with 10% FCS was added to the bottom of 24-well plates. Cells were seeded at a density of 50 000 cells well^−1^ into the upper inserts and incubated at 37°C. After 24 h, the non-invading cells were removed from the upper surface of the separating membrane by gentle scrubbing with a cotton swab. Invading cells were fixed in cold 100% methanol and stained with 0.05% crystal violet in 20% ethanol. The membranes were mounted on glass slides and manually counted using a light microscope. The invasion index was calculated as the percentage of invaded cells in the treatment group compared to the control group. All assays were performed in triplicate.

### Statistical analysis

Results are expressed as the mean±s.e.m. unless indicated otherwise. For statistical analysis, the non-parametric Mann–Whitney test was used for all experiments. Significance was defined as *P*<0.05.

## RESULTS

### Runx2 expression and localisation in pancreatic tissues

**Figure 1:**
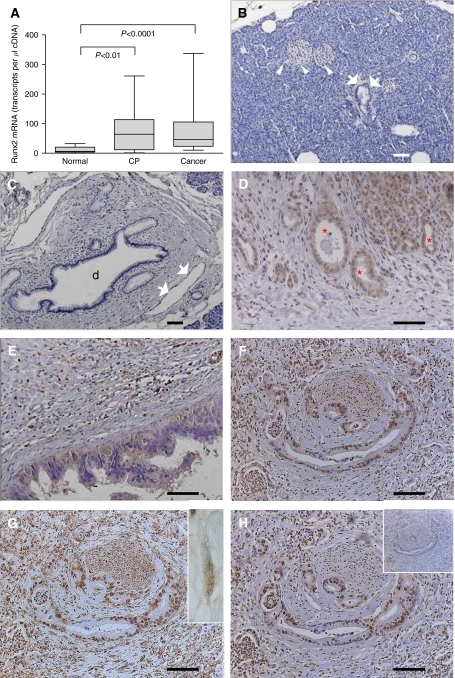
(**A**) Runt-related transcription factor-2 mRNA expression levels in pancreatic tissues: QRT–PCR analysis of mRNA levels for Runx2 in normal, CP, and PDAC tissue samples was carried out as described in the Patients and Methods section (box and whiskers graph). RNA input was normalised to the average expression of the two housekeeping genes HPRT and cyclophilin B, and is presented as transcripts/*μ*l cDNA. (**B–H**) Runx2 localisation in human pancreatic tissues: Runx2 immunohistochemistry was performed as described in the Patients and Methods section. Normal pancreatic tissues showing absent Runx2 staining in the islets (**B**, white arrowheads), acini, and small ducts (**B**, white arrows) as well as large ducts (**C**, d) and endothelial cells (**C**, white arrows). (**D**) Chronic pancreatitis tissues displaying moderate staining in tubular complexes (red asterisks) and degenerating acini. (**E**–**H**) Pancreatic cancer tissues showing moderate Runx2 staining in the cytoplasm of PanIN lesions (**E**), in the cytoplasm and nuclei of cancer cells using three different Runx2 antibodies (**F**–**H**), and fibroblasts (**G**, inset, white arrows). Note the absent staining in a consecutive tissue section incubated with the corresponding normal IgG as a negative control (**H**, inset). Horizontal lines represent the scale bar of 50 *μ*m.

To exactly quantify the mRNA levels of Runx2, Quantitative real-time polymerase chain reaction (QRT–PCR) was carried out in bulk pancreatic tissues. This analysis demonstrated 10.7- and 6.1-fold increases in median Runx2 mRNA levels in CP (*P*<0.01) and PDAC (*P*<0.0001) tissues, respectively, compared to the normal pancreas (Figure 1A). There was a 1.8-fold increase in median Runx2 mRNA levels in CP compared to PDAC, but this was not significant (*P*=0.7). Correlation analysis of Runx2 mRNA expression with TNM staging of PDAC patients revealed a tendency for decreased median Runx2 mRNA levels in PDAC patients with lymph node metastasis compared to PDAC patients without lymph node metastasis (*P*=0.7; data not shown). There was no correlation with other histopathological parameters. To localise Runx2, immunohistochemistry was performed on pancreatic tissue sections from normal (*n*=10), CP (*n*=5) and PDAC (*n*=20) cases. Runt-related transcription factor-2 expression was weak to absent in normal pancreatic tissues. Specifically, acinar and ductal cells were devoid of Runx2 expression (Figure 1B and C). In contrast, in CP tissues there was moderate staining of the tubular complexes (Figure 1D). Moderate Runx2 staining was also observed in PanIN lesions (Figure 1E). Pancreatic cancer cells were positive for Runx2 in 10 out of 20 cases and demonstrated both cytoplasmic and nuclear staining (Figure 1F–H). Additionally, there was moderate-to-strong Runx2 expression in the fibroblasts within the desmoplastic stroma surrounding the cancer cells in 11 out of 20 cases (Figure 1G inset). Three Runx2 antibodies were used in consecutive pancreatic tissue sections to confirm the specificity of Runx2 immunoreactivity (Figure 1F–H). The specificity of the staining was further confirmed using the corresponding normal IgGs as negative controls (Figure 1H inset).

### Effects of coculture between IPSCs and Panc-1 cells

**Figure 2:**
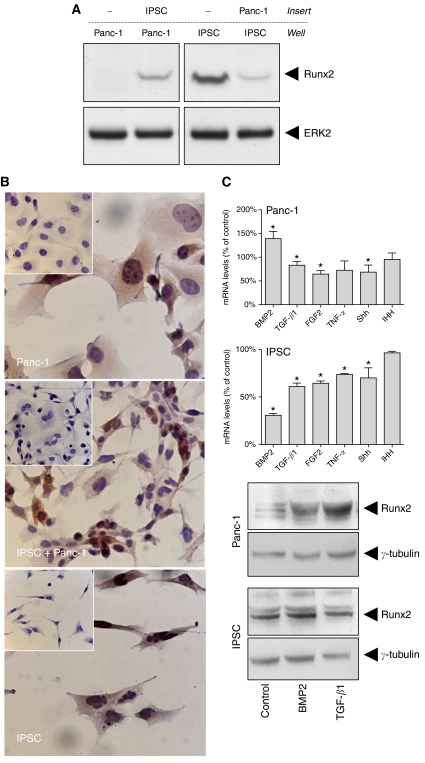
(**A** and **B**) Effects of coculture of IPSCs and Panc-1 cells: (**A**) Cells were cultured in a two-chamber system without cell contact as described in the Patients and Methods section. Cell lysates were obtained from the cells cultured in the wells and immunoblotting was carried out using a Runx2 antibody and an ERK2 antibody as a loading control. (**B**) Cells were cultured alone or cocultured, and immunocytochemistry was performed as described in the Patients and Methods section. Insets represent negative controls incubated with the corresponding normal IgG. (**C**) Effects of growth factors and cytokines on Runx2 mRNA and protein expression in Panc-1 cells and IPSCs: cells were treated with the indicated factors for 48 h. Cell lysates were collected for RNA and protein extraction as described in the Patients and Methods section. Bars represent Runx2 mRNA expression levels as percentage of untreated cells as determined by QRT–PCR. Data are presented as mean±s.e.m. of three independent experiments (^*^P<0.05). Runt-related transcription factor-2 protein expression was determined by immunoblotting (first and third panels). Equal loading was determined using a goat polyclonal *γ*-tubulin antibody (second and fourth panels).

As Runx2 was localised in the cancer cells and the associated fibroblasts in PDAC tissues, next the effects of coculture with or without direct cell-to-cell contact between Panc-1 cells and IPSCs were analysed. Coculture experiments without cell-to-cell contact revealed that soluble factors released from IPSCs were able to increase Runx2 expression in Panc-1 cells. In contrast, factors released from Panc-1 cells reduced Runx2 expression in IPSCs (Figure 2A). On the other hand, coculture with cell-to-cell contact between Panc-1 cells and IPSCs led to no significant difference in the Runx2 staining intensity in Panc-1 cells or IPSCs as shown by immunocytochemistry (Figure 2B).

### Regulators of Runx2 expression in IPSCs and Panc-1 cells

Since Runx2 expression is regulated by factors released from both pancreatic cancer cells and pancreatic stellate cells, we sought to analyse the effects of different growth factors and cytokines on Runx2 expression in these cells (Figure 2C). Interestingly, members of the TGF-*β* family (TGF-*β*1 and BMP2) divergently regulated Runx2 mRNA expression in Panc-1 cells and IPSCs. Thus, TGF-*β*1 led to a mild reduction of Runx2 mRNA expression in Panc-1 cells and IPSCs. In contrast, BMP2 increased Runx2 mRNA expression in Panc-1 cells but decreased Runx2 mRNA expression in IPSCs (Figure 2C). Immunoblotting was carried out to confirm these results at the protein level. Interestingly, both TGF-*β*1 and BMP2 increased Runx2 protein levels in Panc-1 cells (Figure 2C). In contrast, there was no significant change in Runx2 protein levels after treatment of IPSCs with TGF-*β*1 or BMP2. Additionally, there was a significant reduction of Runx2 mRNA expression in Panc-1 cells after FGF2 treatment by −35.7±4.2% and Shh by −31.3±8.5% (Figure 2C). In IPSCs, there was also a significant reduction of Runx2 mRNA expression after treatment with FGF2 by −35.7±1.4%, TNF-*α* by −26.3±0.5%, and Shh by −30±6.1% (Figure 2C).

### Transcriptional targets of Runx2 in IPSCs and Panc-1 cells

**Figure 3:**
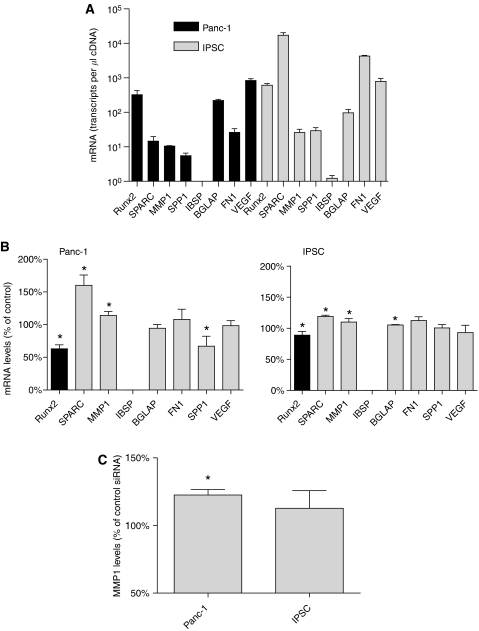
(**A**) mRNA levels of Runx2 and Runx2 target genes in Panc-1 and IPSCs were determined by QRT–PCR as described in the Patients and Methods section and presented as mean±s.e.m. (*n*=3). (**B**) The effects of Runx2 silencing on the mRNA expression of target genes were determined in Panc-1 cells and IPSCs by QRT–PCR analysis and presented as mean±s.e.m. (*n*=3), compared to control transfected cells. (**C**) The effects of Runx2 silencing on MMP1 protein levels were determined by an ELISA assay as described in the Patients and Methods section, and presented as mean±s.e.m. (*n*=3) compared to control transfected cells (^*^P<0.05).

**Figure 4:**
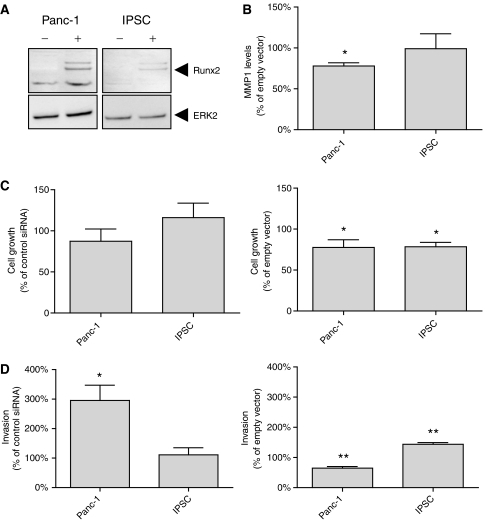
(**A** and **B**) Panc-1 cells and IPSCs were transiently transfected with a full-length Runx2 expression vector or an empty control vector. (**A**) Immunoblot analysis of control-transfected (−) and Runx2 transfected (+) cells was carried out as described in the Patients and Methods section. An ERK2 antibody was used as a loading control. (**B**) MMP1 levels in Runx2 transfected Panc-1 cells and IPSCs were determined using an ELISA assay and are presented as mean±s.e.m. (*n*=3) compared to control transfected cells. (**C** and **D**) The effects of Runx2 silencing (left panel) and Runx2 overexpression (right panel) on cell growth (**C**) and cell invasion (**D**) were determined as described in the Patients and Methods section and are presented as mean±s.e.m. (*n*=3) compared to the respective controls (^*^P<0.05; ^**^P<0.005).

The basal mRNA expression levels of Runx2 and target genes were determined in Panc-1 cells and IPSCs by QRT–PCR (Figure 3A). In the next set of experiments, the transcriptional activity of Runx2 was analysed in IPSCs and Panc-1 cells. Runt-related transcription factor-2 silencing was carried out using specific Runx2 siRNA molecules, resulting in reduction of Runx2 mRNA levels by −37±6% in Panc-1 cells and −11±6% in IPSCs (Figure 3B). There was a significant increase in SPARC and MMP1 mRNA levels in Panc-1 cells of 60±9.2 and 14±4.2%, respectively. In IPSCs, there was a significant increase in SPARC and MMP1 mRNA levels by 19±1.2 and 10±3.2% respectively. At the protein level, there was a significant upregulation of SPARC (data not shown) and MMP1 (Figure 3C) following Runx2 silencing in the cell culture supernatant of Panc-1 cells, but these changes were not significant for IPSCs. The transcriptional activity of Runx2 was analysed after transient Runx2 overexpression in Panc-1 cells and IPSCs using a full-length expression vector (Figure 4A). Following Runx2 overexpression, a significant reduction (−22.1±2.8%) in MMP1 protein levels was detected in the supernatant of Panc-1 cells (Figure 4B). Since the basal SPARC mRNA levels were barely detectable in Panc-1 cells (Figure 3A), the changes in SPARC protein expression following Runx2 overexpression were not detectable by immunoblotting (data not shown). IPSCs exhibited no significant change in MMP1 protein levels by ELISA (Figure 4B), and SPARC by immunoblotting (data not shown). In addition, Runx2 silencing led to a significant reduction of SPP mRNA expression in Panc-1 cells by −33±8.8%, and to a slight increase of BGLAP mRNA levels by +5.3±0.4% in IPSCs (Figure 3B).

### Effects of Runx2 on cell morphology, growth, and invasion of IPSCs and Panc-1 cells

To examine the effects of Runx2 on cell morphology, growth, and invasion of IPSCs and Panc-1 cells, cytology, growth, and invasion assays were performed after Runx2 silencing and Runx2 overexpression. There was no significant change in the cell morphology of IPSCs and Panc-1 cells (data not shown). Both Panc-1 and IPSCs also exhibited no significant change in cell growth after Runx2 silencing. In contrast, Runx2 overexpression led to a significant reduction in cell growth by –22.4±5.4% in Panc-1 cells and −21.5±3.1% in IPSCs (Figure 4C). In the next set of experiments, the effects of Runx2 on the invasion of IPSCs and Panc-1 cells were tested. Runt-related transcription factor-2 silencing led to a significant increase in the invasion of Panc-1 cells by+195±21% compared to controls (Figure 4D). Immortalised pancreatic stellate cells exhibited no significant change in cell invasion after Runx2 silencing. In contrast, Runx2 overexpression led to a significant reduction in cell invasion of Panc-1 by −35.1±2.1%, but increased invasion of IPSCs by 43.8±2.3% (Figure 4D).

## DISCUSSION

Runt-related transcription factor-2 expression is deregulated in many glandular tumours, such as prostate cancer (Brubaker *et al*, 2003; Pratap *et al*, 2005) and breast cancer (Barnes *et al*, 2003). Runx3, a member of the same family, is overexpressed in bulk PDAC tissues (Li *et al*, 2004a), but almost absent in a large group of cultured pancreatic cancer cell lines due to promoter hyper-methylation (Wada *et al*, 2004; Li *et al*, 2004a). In the present study, the weak-to-absent Runx2 staining in the normal pancreas matched the low Runx2 mRNA levels and reflects the homogenous tissue composition (mainly acinar cells). On the other hand, the wide range of Runx2 mRNA expression in PDAC and CP reflects the heterogeneous composition of these tissues, especially of the Runx2 overexpressing tissue elements such as tubular complexes, PanIN lesions, and cancer cells (in PDAC tissues). Interestingly, Runx2 was also localised in the fibroblasts in CP and PDAC, which is in agreement with the relatively high Runx2 mRNA levels observed in cultured IPSCs.

The expression of Runx2 in the cancer cells and the associated stromal fibroblasts in pancreatic cancer tissues prompted us to test the effects of coculture of IPSCs and Panc-1 cells on Runx2 expression as a naturally occurring process *in vivo*. Coculture of both cell lines without a direct cell-to-cell contact led to increased Runx2 expression in pancreatic cancer cells. In contrast Panc-1 cells decreased Runx2 expression in IPSCs. To identify the factors responsible for these effects, various growth factors and cytokines were analysed. Among the analysed factors BMP2 increased Runx2 mRNA levels in Panc-1 cells and both BMP2 and TGF-*β*1 increased Runx2 protein levels in the same cell line. In contrast, BMP2 and TGF-*β*1 reduced Runx2 mRNA levels in IPSCs, but had no apparent effects on Runx2 protein levels. The reasons for the divergent response with respect to Runx2 mRNA and protein levels are not known. However, it has been shown that Runx2 expression can be modulated in several ways, including direct stimulation of gene expression, post-translational modification, and protein–protein interactions (Li *et al*, 2004b; Bae and Lee, 2006; Shen *et al*, 2006). It is known that Runx2 mRNA can be translated by cap-dependent and cap independent internal ribosomal entry site (IRES) mechanisms (Xiao *et al*, 2003; Elango *et al*, 2006; Nishio *et al*, 2006). IRES-dependent translation adds an additional level at which expression of this gene is regulated. The two translational mechanisms can provide the flexibility needed for production of the protein in the appropriate amount at the proper time and in the right cell type as has been shown for Runx1 (Pozner *et al*, 2000; Levanon and Groner, 2004). Therefore, Runx2 translation through different mechanisms might explain the discrepancy between Runx2 protein and mRNA expression levels after TGF-*β*1 or BMP2 treatment. Irrespectively, our results show that cancer and/or stromal cells-derived factors like TGF-*β*1 and BMP2 have the capacity to modulate Runx2 expression in pancreatic cancer and stellate cells in an autocrine/paracrine fashion.

Interestingly, forced Runx2 overexpression in Panc-1 cells reduced cell growth and invasion, whereas Runx2 silencing increased invasiveness in this cell line. It is therefore tempting to speculate that Runx2 – induced via paracrine effects of the activated stroma – functions as tumour suppressive. This is also indirectly supported by the observation that FGF2 and Shh, which are known to increase pancreatic cancer cell growth and invasion (Kornmann *et al*, 1998; Thayer *et al*, 2003), led to reduction of Runx2 mRNA expression in Panc-1 pancreatic cancer cells. In contrast, Runx2 silencing did not affect growth or invasion of IPSCs, likely because of the low efficiency of Runx2 silencing in this cell line. However, Runx2 overexpression led to reduced growth but increased invasion of IPSCs. This phenomenon has also been observed in hepatic stellate cells (HSCs) during tissue remodelling under the effects of another gene, fibroblast activation protein (FAP) (Wang *et al*, 2005). Fibroblast activation protein overexpression in the human HSCs leads to increased cell adhesion, migration, and invasion. Thus, it might be speculated that Runx2 facilitates tissue remodelling via direct or indirect increase in invasion and decrease in the growth of IPSCs, as it has been suggested for FAP in HSCs (Wang *et al*, 2005).

The transcriptional activity of Runx2 target genes was studied in IPSCs and Panc-1 pancreatic cancer cells. In our study, there was low efficiency of Runx2 silencing, although the current protocol used for siRNA transfection has been used for other genes (Berberat *et al*, 2005; Erkan *et al*, 2005; Kayed *et al*, 2005). Furthermore, two additional Runx2 siRNA sequences were used, and these exhibited even lower silencing efficiencies than the one that was used in the final experiments. It is known that the siRNA-based technology displays a wide range of activities, thus reducing target mRNA or protein expression by 0–>90%. Thus, only a fraction of siRNAs result in a significant reduction of targets (Hamada *et al*, 2002; Holen *et al*, 2002), which may be due to several factors. First, the nature of siRNA and the presence of nucleotide mismatch (Elbashir *et al*, 2001; Holen *et al*, 2002; Amarzguioui *et al*, 2003; Czauderna *et al*, 2003). Second, extensive secondary structure in the mRNA, such as the presence of RNA-associated proteins and the specific subcellular localisation of the target mRNA that render the transcript inaccessible to siRNA-incorporated RISC binding (Holen *et al*, 2002). Third, the turnover rate of mRNA and the properties of the target cells, such as variations in transfection efficiency, cell confluence, passage number, differentiation status, and the toxicity of the transfection reagents, all play a role in determining the outcome of gene silencing by siRNA (Hu *et al*, 2004).

Despite the relatively low efficiency of Runx2 silencing in the tested cell lines, there was a significant change in the expression of several target genes such as SPARC and MMP1 in IPSCs and Panc-1 pancreatic cancer cells. SPARC is markedly overexpressed and localised in fibroblasts and extracellular matrix surrounding tumour cells in PDAC, and increases the invasiveness of pancreatic cancer cells (Guweidhi *et al*, 2005). Additionally, SPARC enhances pancreatic tumour growth in SPARC-null mice, where it is associated with decreased deposition of extracellular matrix and reduced cancer cell apoptosis (Puolakkainen *et al*, 2004). Runt-related transcription factor-2 silencing increased SPARC levels in Panc-1 cells. Therefore, the observed low-to-absent SPARC expression levels in some pancreatic cancer cells *in vivo* might be due to suppressive effects of Runx2. Since SPARC is thought to act as a tumour promoter, these findings point again to Runx2 as a potential tumour suppressor. In line with these findings, Runx2 silencing also increased the release of MMP1 from Panc-1 cells, whereas Runx2 overexpression decreased MMP1 levels in the same cell line. These data are in agreement with the known tumour suppressor function of members of the Runt family of transcription factors. Thus, Runx2 and Runx3 might act as tumour suppressors in malignant melanoma (Martinez *et al*, 2005), and Runx3 in breast, gastric, colon, and hepatocellular carcinomas, as well as non-small cell lung cancer (Goel *et al*, 2004; Sakakura *et al*, 2005; Lau *et al*, 2006; Miyagawa *et al*, 2006; Yanagawa *et al*, 2007). The reason why the potential tumour suppressor Runx2 is overexpressed in some pancreatic cancer tissues is currently not known, and requires further studies. Interestingly, increased expression of Runx3 has also been observed in approximately one-third of pancreatic cancer cases (Li *et al*, 2004a). It could be speculated that in the Runx2 or Runx3 overexpressing tumours, other factors might exert stronger tumour promoting effects, and thereby mask the tumour suppressive effects of Runx2 or Runx3.

In conclusion, Runx2 is upregulated in a subset of PDAC tissues, both in tumour cells and the associated fibroblasts. Regulation of Runx2 expression might occur via paracrine mechanisms involving secretion of growth factors such as TGF-*β*1, BMP2, and FGF2 and cytokines such as Shh. Runx2 – in turn – suppresses the transcription of extracellular matrix modulators such as SPARC and MMP1, and thereby influences the pancreatic cancer microenvironment.
